# Neonatal nephron loss during active nephrogenesis – detrimental impact with long-term renal consequences

**DOI:** 10.1038/s41598-018-22733-8

**Published:** 2018-03-14

**Authors:** Carlos Menendez-Castro, Dörte Nitz, Nada Cordasic, Jutta Jordan, Tobias Bäuerle, Fabian B. Fahlbusch, Wolfgang Rascher, Karl F. Hilgers, Andrea Hartner

**Affiliations:** 1 Departments of Pediatrics and Adolescent Medicine, University of Erlangen-Nurnberg, Erlangen, Germany; 2 Departments of Nephrology and Hypertension, University of Erlangen-Nurnberg, Erlangen, Germany; 3 Preclinical Imaging Platform Erlangen, Department of Radiology, University of Erlangen-Nurnberg, Erlangen, Germany

## Abstract

Neonatal nephron loss may follow hypoxic-ischemic events or nephrotoxic medications. Its long-term effects on the kidney are still unclear. Unlike term infants, preterm neonates less than 36 weeks gestational age show ongoing nephrogenesis. We hypothesized that nephron loss during nephrogenesis leads to more severe renal sequelae than nephron loss shortly after the completion of nephrogenesis. Rats show nephrogenesis until day 10 of life resembling the situation of preterm infants. Animals were uninephrectomized at day 1 (UNX d1) resulting in nephron reduction during nephrogenesis and at day 14 of life (UNX d14) inducing nephron loss after the completion of nephrogenesis. 28 days after uninephrectomy the compensatory renal growth was higher in UNX d1 compared to UNX d14. Nephrin was reduced and collagen deposition increased in UNX d1. At 1 year of age, glomerulosclerosis and markers of tubulointerstitial damage were most prevalent in UNX d1. Moreover, the number of desmin-positive podocytes was higher and nephrin was reduced in UNX d1 indicating podocyte damage. Infiltration of inflammatory cells was heightened after UNX d1. Uninephrectomized animals showed no arterial hypertension. We conclude that neonatal nephron loss during active nephrogenesis leads to more severe glomerular and tubulointerstitial damage, which is not a consequence of compensatory arterial hypertension.

## Introduction

Low nephron number is a risk factor for an adverse outcome after renal damage. Clinical and animal studies revealed that an early loss of nephrons during the neonatal period is particularly detrimental to renal outcome^1,2^. Moreover it could be shown that nephron number at birth and the development of chronic kidney disease as well as cardiovascular disease later in life correlate inversely^3–5^. In this context Brenner *et al*. postulated that in individuals with a reduced nephron number sufficient glomerular ultrafiltration is achieved by compensatory glomerular hyperperfusion based on secondary arterial hypertension^6^. In the long term this chronic mechanical burden leads to manifest glomerular and tubulointerstitial damage. Chronic arterial hypertension in turn induces pathologies of the cardiovascular system and the kidneys^7,8^. On the other hand, a reduced nephron number does not necessarily result in hypertension^9^. Thus, the interdependency of reduced renal mass, the development of hypertension and renal damage still remains unclear.

Preterm birth is associated with a higher morbidity and mortality due to the immaturity and higher vulnerability of organs^10^. In contrast to term infants, preterm infants prior to 36 weeks of gestation reveal a still ongoing nephrogenesis^11^. At the same time, these individuals are highly susceptible to renal injury and concomitant nephron loss: Moreover, preterm infants more frequently suffer from neonatal morbidities like neonatal sepsis or patent ductus arteriosus. Acute kidney injury and nephron loss may occur during hypoxic-ischemic events as a consequence of cardiocirculatory decompensation or as side-effects of necessary medications (e.g. antibiotics, non-steroidal anti-inflammatory drugs) frequently resulting in a reduced number of functional nephrons^12^.

In rats a physiologically active nephrogenesis persists until day 10 of life, resembling the renal situation of preterm infants^13^. Uninephrectomy guarantees a defined nephron reduction and induces acute and chronic compensatory mechanisms in the remaining kidney. In this study we tested the hypothesis that acute nephron loss during active nephrogenesis leads to different adaptive changes in the contralateral kidney compared to a nephron loss immediately after termination of nephrogenesis. We further hypothesized that a nephron loss during ongoing nephrogenesis might predispose to more severe renal damage later in life.

## Results

In an animal model of neonatal unilateral nephrectomy (UNX) at day 1 (ongoing nephrogenesis) and at day 14 (nephrogenesis terminated) of life, renal alterations were studied 4 weeks after UNX intervention and at 1 year of age.

### Renal adaptations 4 weeks after UNX

**Figure 1:**
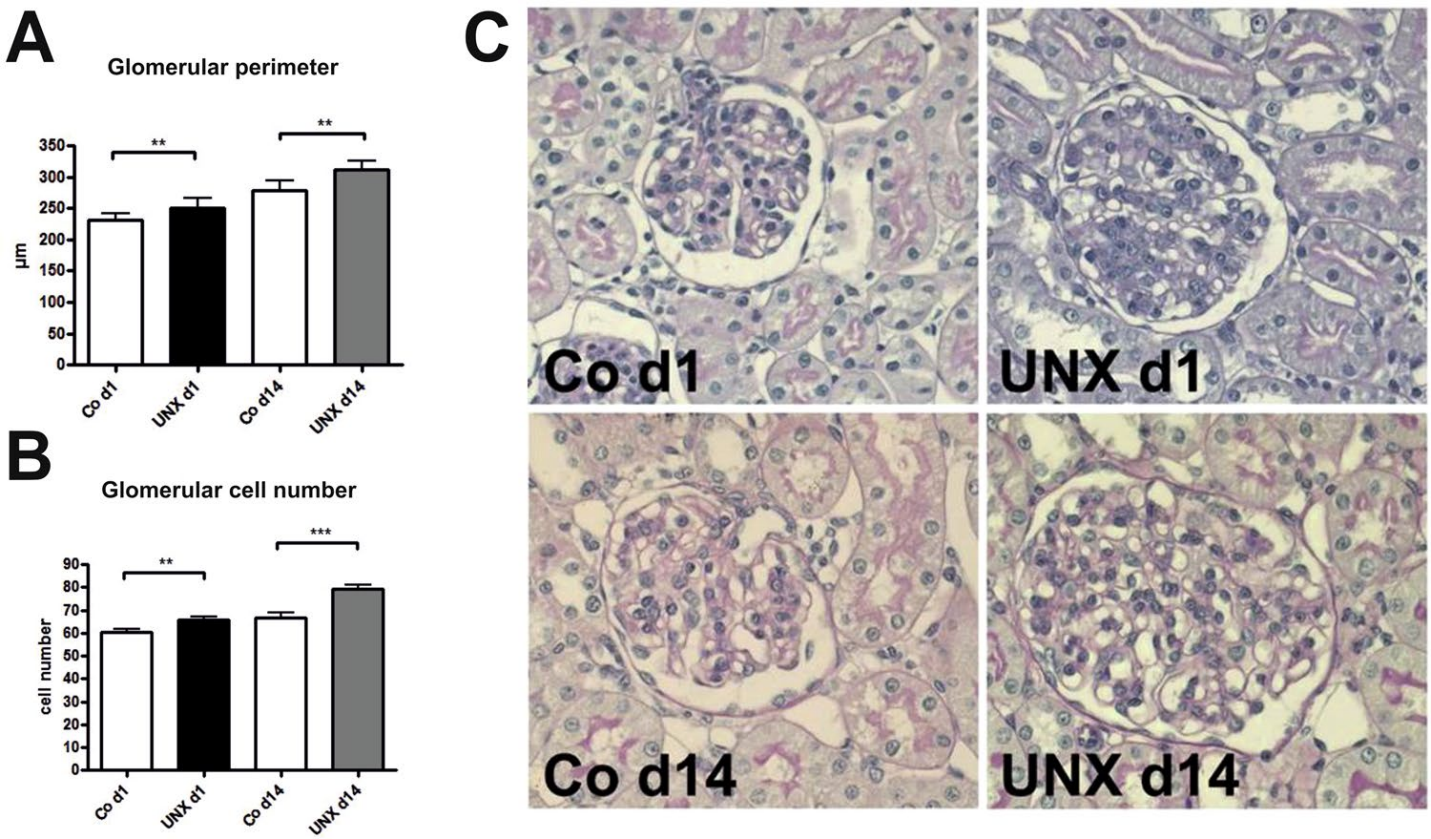
Glomeruli 28 days after uninephrectomy (UNX). (**A**) Perimeter of glomerular cross sections. (**B**) Cell number of glomerular cross sections. (**C**) Representative photomicrographs of PAS-stained renal tissue. UNX d1, uninephrectomy at day 1 of life with respective controls (Co d1). UNX d14, uninephrectomy at day 14 of life with respective controls (Co d14). n = 9 for UNX d1 and n = 9 for respective controls, n = 10 for UNX d14 and n = 6 for respective controls. Data are mean ± SEM. **p < 0.01, ***p < 0.001 (Student’s t-test).

**Figure 2:**
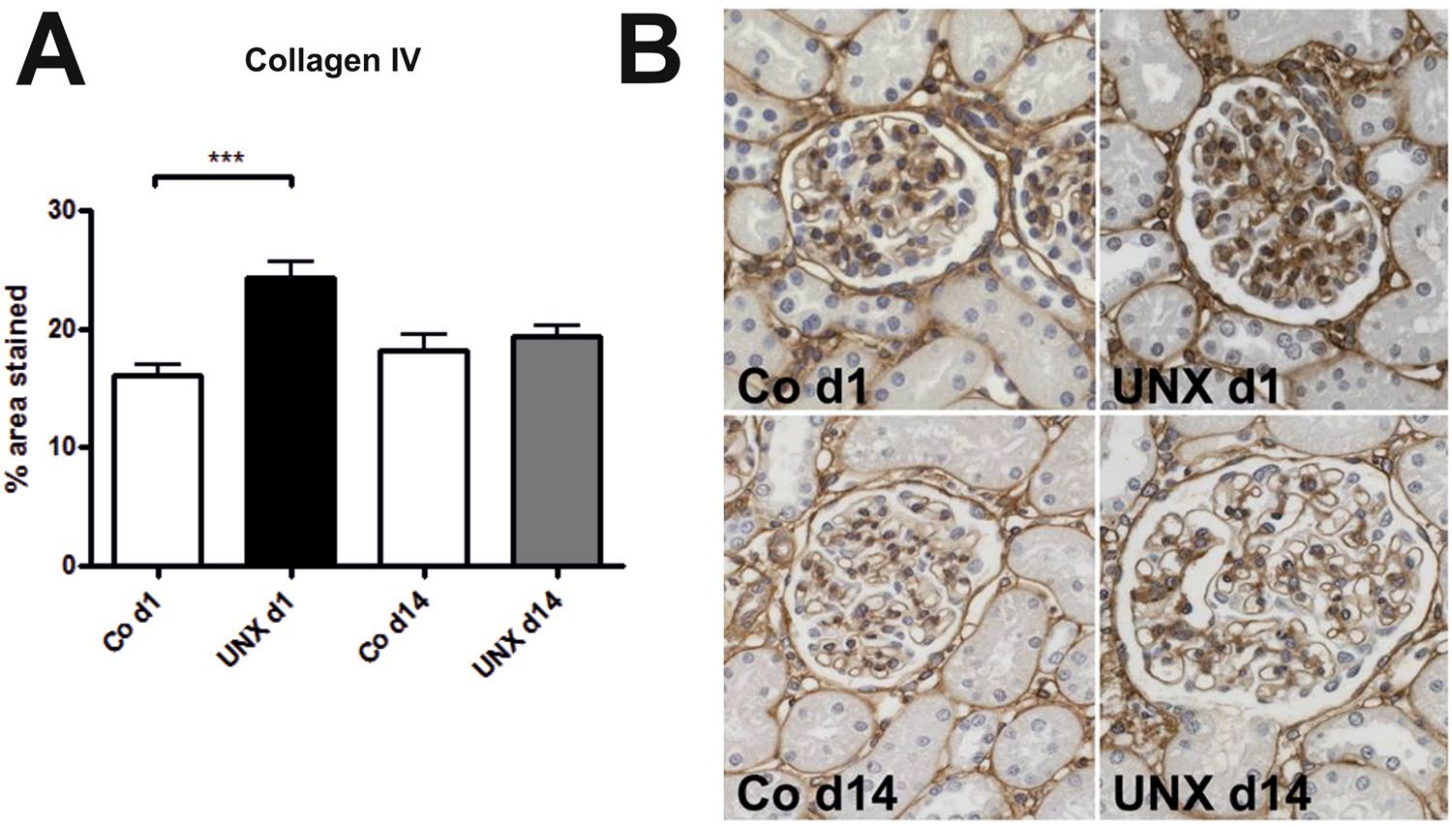
Glomerular collagen IV deposition 28 days after uninephrectomy (UNX). (**A**) Computer-assisted quantification after immunohistochemical detection of collagen IV. (**B**) Representative photomicrographs of collagen IV-stained renal tissue. UNX d1, uninephrectomy at day 1 of life with respective controls (Co d1). UNX d14, uninephrectomy at day 14 of life with respective controls (Co d14). n = 8 for UNX d1 and n = 8 for respective controls, n = 10 for UNX d14 and n = 6 for respective controls. Data are mean ± SEM. ***p < 0.001 (Student’s t-test)

**Figure 3:**
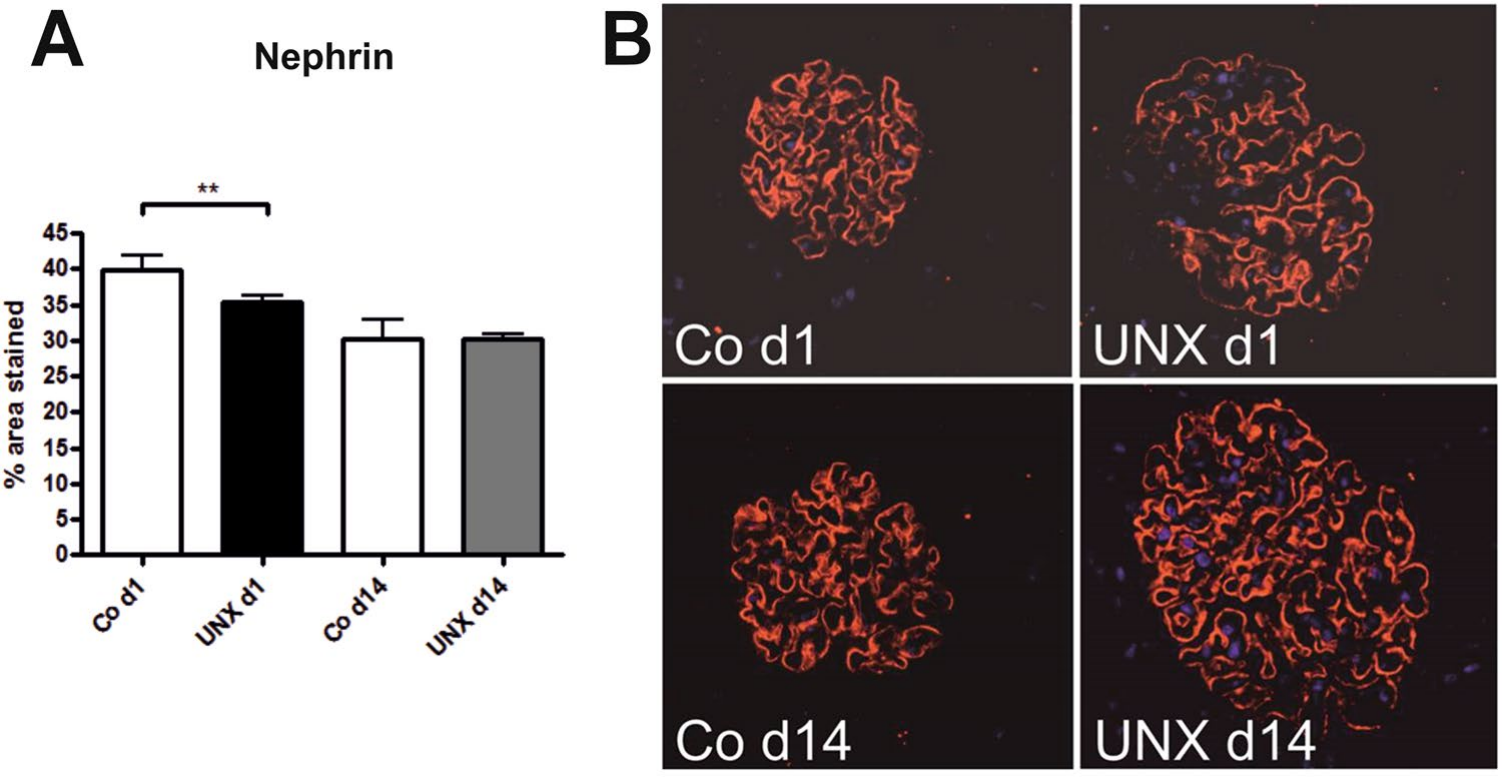
Detection of nephrin in glomeruli 28 days after uninephrectomy (UNX). (**A**), Computer-assisted quantification after immunohistochemical detection of nephrin. (**B**) Representative photomicrographs of nephrin-stained renal tissue. UNX d1, uninephrectomy at day 1 of life with respective controls (Co d1). UNX d14, uninephrectomy at day 14 of life with respective controls (Co d14). n = 9 for UNX d1 and n = 9 for respective controls, n = 9 for UNX d14 and n = 6 for respective controls. Data are mean ± SEM. ** p < 0.01 (Student’s t-test).

Body weights and heart weights were not influenced by UNX (Table 1). Four weeks after UNX, a compensatory growth of the remaining kidney was observed in both, rats with UNX at day 1 of life (UNX d1) and rats with UNX at day 14 of life (UNX d14), as shown in Table 1. Total kidney weight was significantly reduced after UNX compared to respective controls only in UNX d14 animals, not in UNX d1 animals, arguing for a higher degree of compensatory renal growth after UNX d1 compared to UNX d14 (Table 1). Glomerular perimeters were augmented in both UNXd1 and UNXd14 animals compared to controls (Fig. 1A). Moreover, there was a 9.5% increase in glomerular cell number after UNX d1 and an 18.4% increase in glomerular cell number after UNX d14 compared to the respective controls (Fig. 1B). Glomerular deposition of collagen IV was increased in UNX d1 compared to controls, but not in UNX d14 (Fig. 2). Nephrin, as a marker of glomerular integrity, was reduced in glomeruli of UNXd1 animals only (Fig. 3).

**Table 1:** Body and organ weights 28 days after uninephrectomy. Data are expressed as mean ± standard error of the mean. n = 9 for UNX d1 and n = 9 for respective controls, n = 10 for UNX d14 and n = 6 for respective controls. p-values were obtained from Student’s t-test.

	Uninephrectomy at day 1 of life (UNX d1)			Uninephrectomy at day 14 of life (UNX d14)		
	control	UNX d1	p-value	control	UNX d14	p-value
Body weight at UNX [g]	6.98 ± 0.37	6.88 ± 0.31	0.84	27.48 ± 0.99	27.95 ± 0.42	0.62
Body weight 28 days after UNX [g]	76.65 ± 4.81	79.05 ± 2.89	0.62	189.03 ± 6.87	190.24 ± 5.11	0.89
Heart weight 28 days after UNX [g]	0.41 ± 0.03	0.41 ± 0.02	0.84	0.74 ± 0.03	0.75 ± 0.01	0.54
Right kidney weight 28 days after UNX [g]	0.49 ± 0.04	0.83 ± 0.04	<0.0001	0.89 ± 0.03	1.28 ± 0.03	<0.0001
Total kidney weight 28 days after UNX [g]	0.96 ± 0.08	0.83 ± 0.04	0.14	1.77 ± 0.04	1.28 ± 0.03	<0.0001
Relative kidney weight 28 days after UNX	1.27 ± 0.06	1.05 ± 0.06	0.018	0.94 ± 0.02	0.68 ± 0.02	<0.0001

### Renal alterations at 1 year of age

At 52 weeks of age body weights were not significantly different between controls, UNX d1 and UNX d14 animals (Table 2). There were no differences in mean arterial blood pressure values between groups (Table 2). Relative left ventricular weights as a marker of myocardial hypertrophy were comparable in all groups (Table 2).

**Table 2:** Renal and cardiovascular parameters at 52 weeks of life. Data are expressed as mean ± standard error of the mean. n = 8 for UNX d1, n = 7 for UNX d14, n = 8 for controls. P-values were obtained from Bonferroni’s post hoc-test following one-way ANOVA or Dunn’s test following Kruskal-Wallis test.

	control	UNX d1	p-value UNX d1 vs control	UNX d14	p-value UNX d14 vs control	**p-value UNXd1**
Body weight [g]	761.2 ± 22.6	743.0 ± 27.6	0.93	775.3 ± 19.7	0.96	0.73
Kidney weight [g]	3.89 ± 0.15	3.49 ± 0.22	0.30	3.21 ± 0.10	0.03	0.81
Relative kidney weight [%]	0.51 ± 0.02	0.46 ± 0.01	0.22	0.41 ± 0.01	0.003	0.16
Glomerular perimeter [µm]	362.1 ± 7.5	413.0 ± 5.7	0.001	411.4 ± 11.3	0.002	0.99
Glom. cell number [no/glom]	43.7 ± 3.4	57.7 ± 5.4	0.08	50.3 ± 3.4	0.86	0.71
PCNA-pos. glomerular cells [no/glom]	0.15 ± 0.04	0.24 ± 0.04	0.65	0.19 ± 0.06	>0.99	>0.99
Relative left ventricular weight [%]	0.172 ± 0.006	0.19 ± 0.01	0.31	0.175 ± 0.004	>0.99	0.52
Mean arterial pressure [mmHg]	118.8 ± 2.1	122.1 ± 4.2	>0.99	117.1 ± 2.9	>0.99	>0.99
Renin mRNA [fold change]	1.00 ± 0.17	0.82 ± 0.14	>0.99	0.95 ± 0.14	>0.99	>0.99
Renin-pos. juxtaglomerular apparatus [%]	4.29 ± 1.09	2.31 ± 0.52	0.21	3.16 ± 0.71	0.78	>0.99
Plasma renin activity [ng AI/ml/h]	4.37 ± 0.75	2.26 ± 0.40	0.026	3.23 ± 0.20	0.44	0.64

In contrast, relative total renal mass was significantly reduced only in UNX d14 animals compared to controls (Table 2). However, relative total renal mass was not significantly different between UNX d1 and UNX d14 (Table 2). Glomerular perimeters were increased in both UNX d1 and UNX d14. Glomerular cell numbers only tended to be increased after uninephrectomy (Table 2). Glomerular cell proliferation was not different in the groups investigated (Table 2). Plasma renin activity was significantly reduced in UNX d1 animals compared to controls but plasma renin activity of UNX d1 was not significantly different from UNX d14 (Table 2). Renin expression and the number of renin-positive juxtaglomerular apparatus tended to be somewhat reduced in UNX d1 (Table 2). In both, UNX d1 and UNX d14 animals, albuminuria was elevated compared to controls (Table 3). Serum urea, serum creatinine and creatinine clearance was not significantly different in the experimental groups (Table 3). The expression of biomarkers of renal injury i.e., kidney injury molecule-1 (KIM-1) and neutrophil gelatinase-associated lipocalin (NGAL), was induced after UNX, but was comparable in the kidneys of UNX d1 and UNX d14, while uromodulin (Umod) expression was not different in the experimental groups (Table 3).

**Table 3:** Markers of kidney injury at 52 weeks of age. Data are expressed as mean ± standard error of the mean. n = 8 for UNX d1, n = 7 for UNX d14, n = 8 for controls. P-values were obtained from Bonferroni’s post hoc-test following one-way ANOVA or Dunn’s test following Kruskal-Wallis test.

	control	UNX d1	p-value UNX d1 vs control	UNX d14	p-value UNX d14 vs control	p-value UNX d1 vs UNX d14
Albuminuria [mg/24 h]	14.95 ± 6.70	338.9 ± 66.4	0.049	248.8 ± 58.2	0.029	>0.99
Serum urea [mg/dl]	34.0 ± 1.57	38.7 ± 2.41	0.38	41.7 ± 2.45	0.07	>0.99
Serum creatinine [mg/dl]	0.309 ± 0.02	0.356 ± 0.02	0.38	0.384 ± 0.02	0.03	0.75
Creatinine clearance [ml/min]	4.60 ± 0.62	4.02 ± 0.30	>0.99	3.96 ± 0.19	0.93	>0.99
KIM-1 mRNA [fold induction]	1.00 ± 0.29	4.51 ± 1.47	0.009	6.74 ± 2.92	0.023	>0.99
NGAL mRNA [fold induction]	1.00 ± 0.10	3.50 ± 0.68	0.006	3.41 ± 0.73	0.016	>0.99
Umod mRNA [fold induction]	1.00 ± 0.18	0.67 ± 0.10	0.20	0.78 ± 0.06	0.66	>0.99
CD2AP mRNA [fold induction]	1.00 ± 0.12	1.41 ± 0.39	0.88	1.25 ± 0.15	>0.99	>0.99
NEPH1 mRNA [fold induction]	1.00 ± 0.09	1.19 ± 0.21	>0.99	1.25 ± 0.09	0.82	>0.99
Podocin mRNA [fold induction]	1.00 ± 0.12	1.08 ± 0.11	0.89	1.25 ± 0.13	0.36	0.58
WT-1 pos. glom. cells [no/glom]	6.48 ± 0.22	7.29 ± 0.32	0.12	6.38 ± 0.25	>0.99	0.10

**Figure 4:**
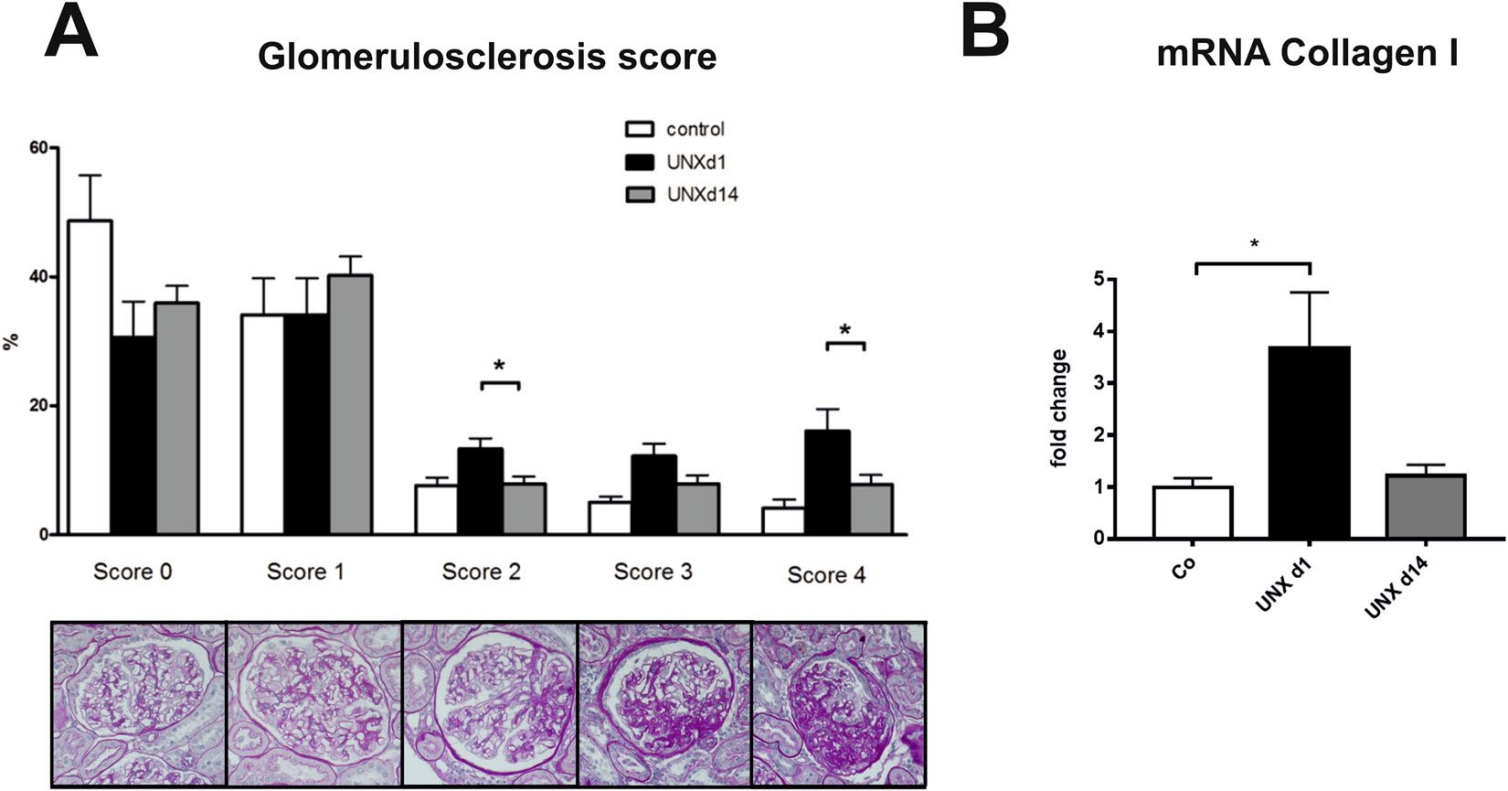
Renal fibrosis at 52 weeks of age. (**A**) Glomerulosclerosis score with representative photomicrographs of PAS-stained glomeruli. (**B**) mRNA expression analysis of collagen I in renal cortical tissue. UNX d1, uninephrectomy at day 1 of life. UNX d14, uninephrectomy at day 14 of life. Co, control group with two kidneys. n = 8 for UNX d1, n = 7 for UNX d14, n = 8 for controls. Data are mean ± SEM. *p < 0.05 (one-way ANOVA, followed by Bonferroni *post hoc* test).

**Figure 5:**
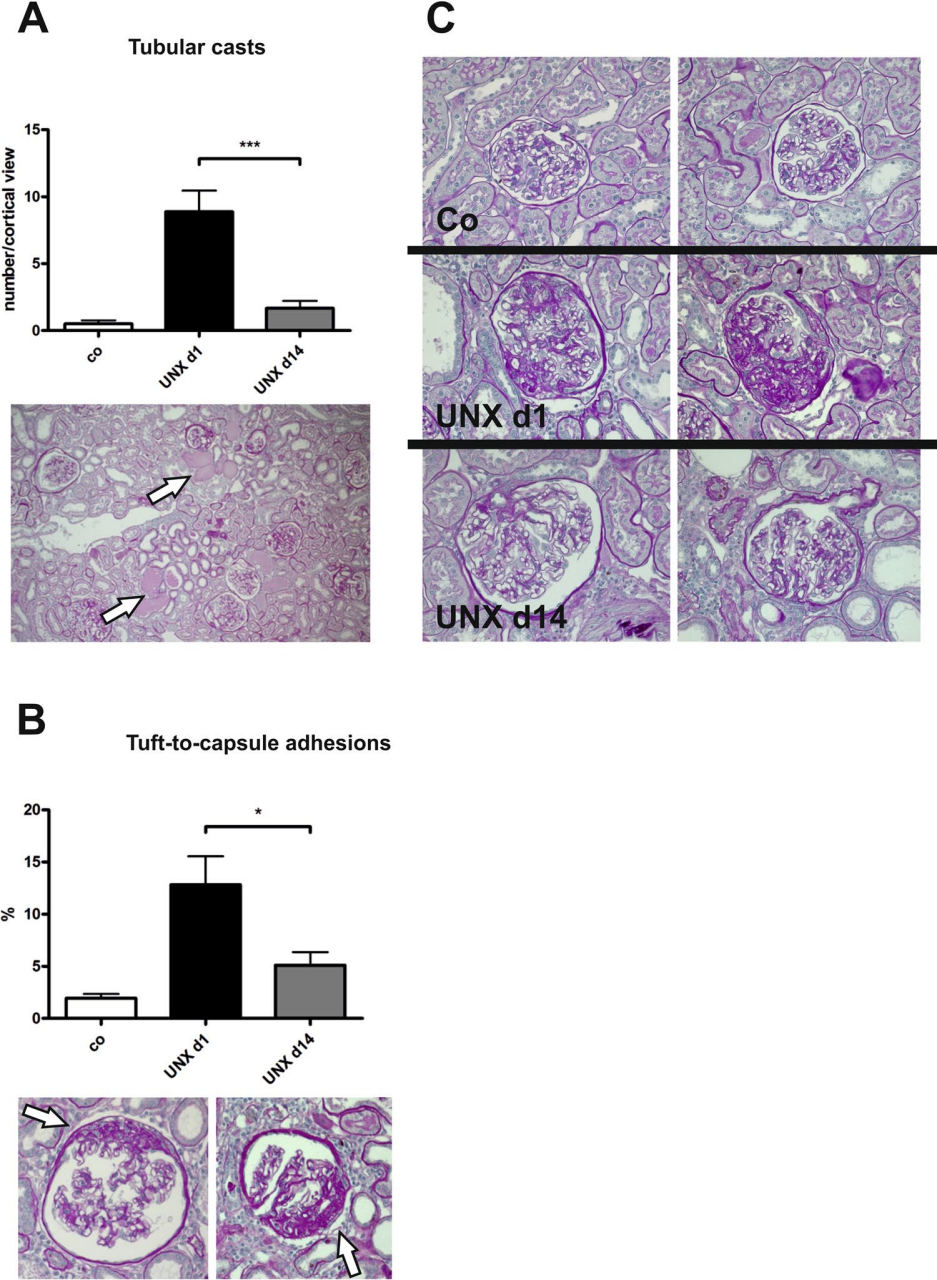
Glomerular tuft-to-capsule adhesions and tubular casts at 52 weeks of age. (**A**) Quantification of the number of tubular casts in PAS-stained renal sections. Arrows point to tubular casts. (**B**) Evaluation of the percentage of glomeruli with tuft-to-capsule adhesions. Arrows point to tuft-to-capsule adhesions. (**C**) Exemplary photomicrographs of glomeruli of control group, UNX d1 and UNX d14 group. UNX d1, uninephrectomy at day 1 of life. UNX d14, uninephrectomy at day 14 of life. Co, control group with two kidneys. n = 8 for UNX d1, n = 7 for UNX d14, n = 8 for controls. Data are mean ± SEM. * p < 0.05, *** p < 0.001 (one-way ANOVA, followed by Bonferroni *post hoc* test).

**Figure 6:**
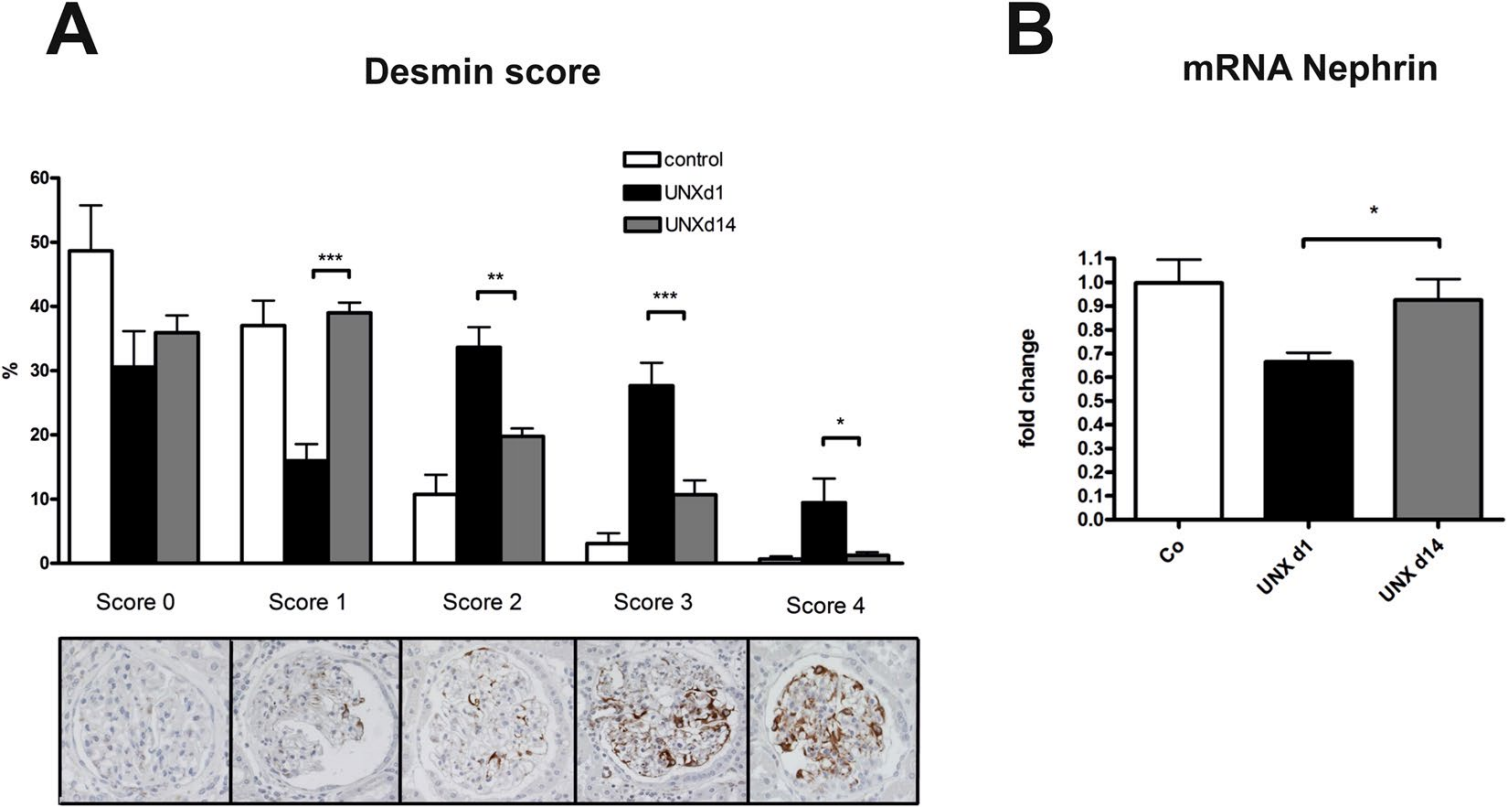
Markers of podocyte damage at 52 weeks of age. (**A**) Glomerular desmin score assessed in PAS-stained renal sections. (**B**) Nephrin mRNA expression in renal cortical tissue. UNX d1, uninephrectomy at day 1 of life. UNX d14, uninephrectomy at day 14 of life. Co, control group with two kidneys. n = 8 for UNX d1, n = 7 for UNX d14, n = 8 for controls. Data are mean ± SEM. * p < 0.05, ** p < 0.01, *** p < 0.001 (one-way ANOVA, followed by Bonferroni *post hoc* test).

On the other hand, renal fibrotic remodelling was significantly more severe in UNX d1 compared to UNX d14, as assessed by a glomerulosclerosis score (Fig. 4A) and by collagen I expression analysis (Fig. 4B). Moreover, tubular casts were more frequent in tubuli of UNX d1 (Fig. 5A) and more glomeruli were affected by tuft-to-capsule adhesions in UNX d1 kidneys (Fig. 5B). This was also reflected by a desmin score, which revealed more podocyte damage in UNX d1 compared to UNX d14 or controls (Fig. 6A). Nephrin as a marker of podocyte integrity showed a significantly reduced renal mRNA expression in UNX d1 animals compared to the other study groups (Fig. 6B), while other markers of intact podocytes, podocin, nephrin-like protein 1 (NEPH1) and CD2-associated protein (CD2AP), were not different in the experimental groups (Table 3). The number of podocytes in UNX d1 and UNX d14, as evaluated by counting Wilm’s Tumor protein 1 (WT-1) positive glomerular cells, did not differ from podocyte numbers of controls (Table 3 and Supplemental Fig. 1). Together with the described increase in glomerular perimeters in UNX d1 and UNX d14 (Table 2), this might argue for increased podocyte stress in both UNX groups.

Evaluation of tubulointerstitial fibrosis (e.g. interstitial collagen deposition or fibroblast activation, vimentin and alpha-smooth muscle actin (α-SMA) expression) did not reveal significant differences in the kidneys of UNX d1 and UNX d14 (Supplemental Table 2). Expression analysis of collagen IV and fibrotic mediators in cortical tissue (plasminogen activator inhibitor 1 (PAI-1), transforming growth factor beta 1 (TGFβ1), TGFβ2, tissue inhibitor of metalloproteinases 2 (TIMP-2), matrix metalloproteinase 2 (MMP-2) did not reveal significant differences in the experimental groups with the exception of tissue inhibitor of metalloproteinases 1 (TIMP-1) expression, which was induced in UNX d1. The expression of fibronectin tended to be somewhat increased in UNX d1 (Supplemental Table 2). Renal capillarization was not affected by uninephrectomy (Supplemental Table 2 and Supplemental Fig. 1). Moreover, the expression of platelet endothelial cell adhesion molecule (PECAM), vascular endothelial growth factor (VEGF) and its receptor as markers of angiogenesis and capillarization was not different in the groups investigated (Supplemental Table 2).

**Figure 7:**
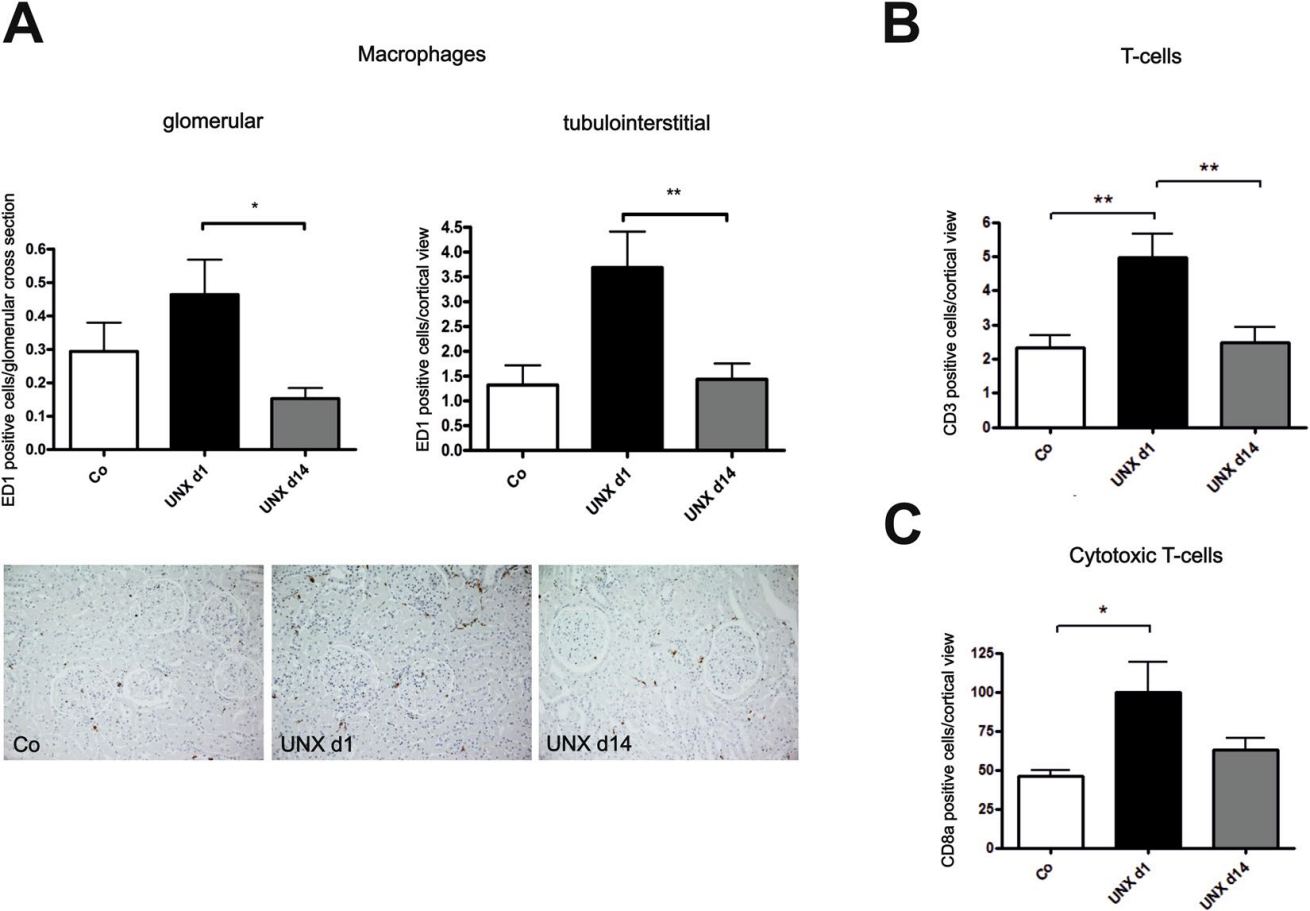
Renal immune cell infiltration at 52 weeks of age. (**A**) Quantification of glomerular and interstitial macrophage infiltration with exemplary photomicrographs of renal tissue stained for ED-1. (**B**) Quantification of tubulointerstitial T-cell infiltration. (**C**) Quantification of tubulointerstitial cytotoxic T-cell infiltration. UNX d1, uninephrectomy at day 1 of life. UNX d14, uninephrectomy at day 14 of life. Co, control group with two kidneys. n = 8 for UNX d1, n = 7 for UNX d14, n = 8 for controls. Data are mean ± SEM. * p < 0.05, ** p < 0.01 (one-way ANOVA, followed by Bonferroni *post hoc* test).

The infiltration of inflammatory cells into the kidney was increased in UNX d1 compared to UNX d14 or controls. The frequency of glomerular and interstitial macrophages (Fig. 7A) as well as cortical T-cells (Fig. 7B) and cytotoxic T-cells (Fig. 7C) was highest in UNX d1. We also assessed the expression of selected cytokines and chemokines. Most of them did not differ significantly in UNX d1 and UNX d14 (Supplemental Table 3). Osteopontin expression, however, was significantly higher in UNX d1 than in controls (Supplemental Table 3), while interleukin-6 (IL-6) and C-C motif chemokine ligand 7 (CCL-7) showed only a tendency to a higher expression in UNX d1 (Supplemental Table 3).

## Discussion

The main finding of this study is that neonatal nephron loss during ongoing nephrogenesis results in more severe glomerular and tubulointersitial damage than nephron loss after termination of nephrogenesis. These renal changes are not a consequence of arterial hypertension. This is in contrast to the findings of Woods *et al*. who described arterial hypertension after neonatal uninephrectomy in male and female pups^4,14^. These divergent results might be explained by the use of different protocols of blood pressure measurements (2 hours vs. 7 days of recovery after catheter implantation), or the use of different rat strains (Wistar vs. Sprague Dawley). Sprague Dawley rats were found to be more likely to develop hypertension than Wistar rats^15^. Woods *et al*. observed an age-dependent increase of arterial hypertension in animals after neonatal uninephrectomy preceding renal damage^4^. Similar findings of increased blood pressure were made in an ovine model of nephron loss during renal development: Female and male sheep developed higher basal blood pressure after fetal uninephrectomy compared to controls and subsequently a decrease in renal function^16–19^. This would argue for different underlying pathomechanisms compared to our study (primary arterial hypertension vs. primary structural changes of the kidney).

There are conflicting data concerning the development of hypertension in humans with reduced renal mass: Our observations of arterial normotension after early uninephrectomy are in line with several clinical studies showing no significantly increased incidence of arterial hypertension in individuals born with a solitary kidney or in kidney donors^20,21^. On the other hand, there are also several clinical studies, which describe an increased risk for the development of hypertension in children with a solitary kidney^5,22^ or in kidney donors^23^. The reasons for the discrepancies in study outcomes regarding blood pressure are still unclear, but might reflect different study designs or differences in the selection of the study collective.

What might account for the differences in renal outcome of rats uninephrectomized during ongoing nephrogenesis versus rats uninephrectomized shortly after the completion of nephrogenesis? We do not have any evidence that uninephrectomy at day 1 of life results in a higher nephron number compared to uninephrectomy later in life (see Supplemental Fig. 2). This is in accordance with findings by Tögel *et al*., who described no detectable de novo nephrogenesis in mice after neonatal uninephrectomy^24^. Therefore, we do not believe that differences in nephron numbers can account for the observed differences in renal outcome in our experimental groups. In contrast, Douglas-Denton *et al*. found a significant increase of nephron number after fetal uninephrectomy in the ovine fetus^25^. Different effects of uninephrectomy might not only be due to species differences, but also to the difference between intrauterine/prenatal vs. extrauterine/postnatal conditions. Nevertheless, we also detected early renal adaptations after uninephrectomy: In both uninephrectomized groups glomerular size and cell number increased. An increase in glomerular size is a common feature in many models of reduced renal mass^26,27^. Acute nephron reduction, e.g. uninephrectomy, commonly results in compensatory renal growth^26^. Compensatory renal growth was, however, most prominent in rats after uninephrectomy during ongoing nephrogenesis. An influence of age on compensatory renal growth was described in several studies. Uninephrectomy at a young age resulted in more compensatory renal growth than uninephrectomy at an older age^28^. In our study, this compensatory renal growth was accompanied by increased glomerular collagen deposition and some loss of podocyte integrity only in rats uninephrectomized during ongoing nephrogenesis. This is in accordance with findings by Okuda *et al*.^29,30^ and Celsi *et al*.^31^, who described a declining incidence of glomerulosclerosis in rats with increasing age at the time of uninephrectomy.

At the age of one year, all rats showed some degree of glomerulosclerosis, with rats uninephrectomized during ongoing nephrogenesis having developed the most prominent fibrotic changes. Moreover, podocyte integrity seemed to be somewhat compromised in rats after uninephrectomy during ongoing nephrogenesis. Nephrin is an important regulator of kidney development mediating podocyte maturation^32^ and a crucial factor to maintain glomerular structure and integrity later in life^33,34^. Glomerular disease is frequently accompanied by an altered expression of nephrin^35^. In our study cortical nephrin expression was significantly reduced in rats uninephrectomized during ongoing nephrogenesis compared to the other experimental groups. Thus, early neonatal uninephrectomy seems to lead to a more severe impact on nephrin-associated regulatory pathways and might enhance the development of primary podocyte damage later in life, as shown by evaluation of desmin as a marker of podocyte damage in our study.

On the other hand, besides a slightly higher albuminuria no significant differences in renal function were yet observed between the two uninephrectomized groups at the age of one year. It is, however, conceivable that rats after uninephrectomy during ongoing nephrogenesis, which display more severe histological renal changes, might be more vulnerable to an additional burden on the kidney. In humans, individuals with a congenital solitary kidney are known to be at a higher risk of developing renal failure later in life than patients uninephrectomized later in life^5^.

The mechanisms resulting in an increased susceptibility for developing kidney disease when renal mass is lost early in life are still unclear. We hypothesized that uninephrectomy during ongoing nephrogenesis might result in an acceleration of capillary loss, which is regularly observed with progressing age^36^. Therefore, we investigated renal capillarization and markers of angiogenesis, but did not detect significant differences in renal capillarization between the experimental groups. On the other hand, animal studies revealed that uninephrectomy was associated with the induction of inflammatory and fibrotic pathways in the remaining kidney^37,38^. In our study, renal inflammation was augmented in rats after uninephrectomy during ongoing nephrogenesis. In the remaining kidney of these animals leukocyte infiltration was most prominent, osteopontin expression was increased and the expression of CCL7 and IL-6 tended to be higher. Triggering pathways of inflammation and fibrosis at this early state of organ plasticity might be the pathogenetic link between early neonatal nephron loss and renal alterations later in life according to the hypothesis of fetal programming. Barker and colleagues postulated that within the period of fetal development external factors can induce structural and functional changes in organs, which in turn supports the development of secondary diseases in adult life^39^.

According to Brenner’s hypothesis, changes of the glomerular architecture and integrity as a consequence of low nephron number occur secondarily following compensatory glomerular hyperfiltration^6^. Concordantly, other studies hypothesized that renal injury after neonatal uninephrectomy is a consequence of arterial hypertension^2,4^. In contrast, our study detected tubulointerstitial and glomerular changes after neonatal uninephrectomy in the absence of arterial hypertension.

After uninephrectomy a dysregulation of the renin-angiotensin system is frequently observed: The activity of angiotensin converting enzyme (ACE) was significantly augmented in rats after uninephrectomy compared to controls, which indicates that activation of RAS might contribute to the maintenance of the homeostasis of glomerular filtration rate after nephron reduction^40^. Moreover, uninephrectomy resulted in a loss of ischemia-induced increase of plasma renin activity^41^. In our study, plasma renin activity was significantly reduced after uninephrectomy in rats with ongoing nephrogenesis only. The observed reduction in renin angiotensin aldosterone system (RAAS) activity, however, was only minor and not associated with overt changes in the animals’ blood pressure. Reduced RAAS activity is a typical feature of the aging kidney^42^. Magro *et al*. described a decrease in plasma renin activity preceding glomerulosclerosis in aging rats^43^. Plasma renin activity was also reduced in long-term observations in sheep after fetal uninephrectomy^44^. This is consistent with our findings of a somewhat decreased plasma renin activity in aging rats after uninephrectomy during ongoing nephrogenesis, which develop more prominent glomerulosclerosis than rats uninephrectomized after completion of nephrogenesis.

Finally, as cardiovascular and renal outcome is known to be sex-dependent, a limitation of our study has to be mentioned: Our study is limited in that we only studied male individuals and therefore cannot appraise the effects of early nephron loss in females, which might differ from the effects seen in males, as reviewed by Lankadeva and colleagues^18^.

Taken together, our data suggests that nephron loss during ongoing nephrogenesis results in glomerular and tubulointerstitial damage in the absence of arterial hypertension later in life. In contrast, a nephron loss after completion of nephrogenesis results in more subtle renal alterations. Our animal model of uninephrectomy during ongoing nephrogenesis or after completion of nephrogenesis reflects the situation of an acute nephron loss in human preterm or term neonates, respectively. Thus, preterm neonates who suffer from medication-induced nephron loss during their neonatal period might be of a particular risk to develop renal disease later in life. Our observations highlight the urgent need to avoid neonatal renal damage with acute nephron loss in preterm infants and may help to develop adapted procedures of prevention and aftercare for this patient cohort.

## Materials and Methods

### Animal procedures

All procedures performed on animals were approved by the local government authorities (Regierung von Mittelfranken, AZ No. 54.2532.1-24/10) and were done in compliance with the DIRECTIVE 2010/63/EU of the European Parliament. Pregnant female Wistar rats were kept on a standard diet (#1320, Altromin, Lage, Germany) with free access to tap water in a room maintained at 22 ± 2 °C with a 12 h dark/light cycle. After spontaneous delivery male pups were divided into two groups: Neonatal left sided uninephrectomy (UNX) was performed on day 1 (UNX d1) and day 14 (UNX d14) of life under isoflurane anesthesia. More detailed information on the periinterventional and surgical procedures is available in the supplementary information (supplemental methods). Histological analyses were performed after 4 weeks of recovery (n = 9 for UNX d1 and n = 9 for respective sham operated controls, n = 10 for UNX d14 and n = 6 for respective sham operated controls) and at the age of one year (n = 8 for UNX d1, n = 7 for UNX d14 and n = 8 for controls). For each group, animals were used from 4 litters, with UNX and controls deriving from the same litters. Controls of the 1 year time point were combined (4 controls for UNX d1 and 4 controls for UNX d14) as they did not differ in their outcome. Animals of each group obtained the standard rat chow (19% protein, 0.24% sodium). One week before sacrifice, the rats were kept in metabolic cages for 24 hours to collect urine. Blood samples were obtained at sacrifice after isoflurane anesthesia. Rats were sacrificed and kidneys and adrenal glands were weighed. For kidney analyses, tissue samples were prepared for histology, RNA isolation and immunohistochemistry as described^45,46^.

### Blood pressure measurements

Blood pressure values were obtained by intra-arterial blood pressure measurements. At the day of sacrifice, rats were anesthetized with isoflurane. Catheters were implanted in the right femoral artery and tunneled subcutaneously. After a recovery phase of 2 hours mean arterial blood pressure was recorded by a polygraph (Hellige, Freiburg, Germany) in conscious animals for 30 minutes.

### Histological analyses

Glomerular cell number and glomerulosclerosis were assessed in kidney sections stained with periodic acid-Schiff’s (PAS) reagent. For glomerular cell number, nuclei were counted in 50 glomeruli per renal cross section. Quantification of glomerulosclerosis was performed by using a semiquantitative score of of 0 to 4 as described before^46^ In short, Score 0 reflects an unaffected glomerulus, score 1 mesangial expansion or sclerosis involving <25% of the glomerular tuft; score 2 equals a sclerosis 25–50%; score 3 represents a sclerosis 50–75%, and/or segmental extracapillary fibrosis or proliferation; score 4 indicates a global sclerosis >75% or global extracapillary fibrosis of proliferation, or complete collapse of the glomerular tuft. All histological evaluations were performed by an investigator blinded to the group assignment.

### Serum analyses

Serum cholesterol, triglycerides, creatinine and urea were measured using an automatic analyser Integra 800 (Roche Diagnostic, Mannheim, Germany). Plasma renin activity was measured with a commercially available radioimmunoassay kit (DiaSorin, Stillwater, MN, USA).

### Immunohistochemistry

For immunohistochemical staining kidneys were fixed in methyl carnoy’s solution and embedded in paraffin. 2 µm sections were stained as described below. Sections were blocked with 3% H_2_O_2_. Primary antibodies were incubated overnight at the following dilutions: Rat endothelial cell antigen (RECA, AbD Serotec, Dusseldorf, Germany) in a dilution of 1:20. Proliferating cell nuclear antigen (PCNA) was used for detection of proliferating cells (M0879; DAKO, Hamburg, Germany) 1:50, ED-1 for macrophages (LMU8949; Linaris, Dossenheim, Germany) 1:50, CD3 for T-cells (I7A2; BioLegend, Fell, Germany) 1:300, CD8a for cytotoxic T-cells (abcam, Cambridge, England) 1:50. Morevover the following antibodies were used at the respective dilutions: Collagen IV (Southern Biotechnology, Birmingham, AL, USA) 1:500, α-SMA (DAKO, Hamburg, Germany) 1:50, desmin (DAKO) 1:50, renin (Proteintech, Manchester, England) 1:100, nephrin (Acris, Hiddenhausen, Germany) 1:50, WT-1 (Santa Cruz Biotech, Heidelberg, Germany) 1:50. Appropriate secondary antibodies (Vector) were diluted 1:500, before avidin D peroxidase (Vector) was applied at a dilution of 1:2000. Finally DAB (Vector) was added, sections were counterstained with hematoxylin and covered with entellan.

WT-1 and PCNA-positive glomerular cells were counted in 50 glomeruli/renal section. RECA-positive capillaries were counted in 20 non-overlapping high-power cortical views. Interstitial smooth muscle actin-positive cells (activated fibroblasts), CD3-positive cells (T-cells), CD8a-positive cells (cytotoxic T-cells) and ED-1-positive cells (macrophages) were counted in 5 non-overlapping medium-power cortical views and presented as positive cells/cortical view. Renin-positive juxtaglomerular apparatus were counted in renal sections and were expressed as % of the total number of glomeruli in a renal section. Glomerular collagen IV expression was quantified in 20 glomeruli/renal section, as a ratio of stained area/total area of glomerular cross sections, using MetaVue software (Molecular devices, Sunnyvale, CA, USA) software.

Glomerular desmin staining in podocytes was assessed using a semiquantitative scoring system as described^47^: Score 0: absent staining or staining less than 5% of the assessed area; Score I: 5–25% stained area; Score II: 25–50% stained area; Score III: 50–75% stained area; Score IV: > 75% stained area. Glomerular desmin expression was quantified in 20 glomeruli/renal section, as a ratio of stained area/total area of glomerular cross sections, using MetaVue software. All immunohistochemical evaluations were done in a blinded manner.

### Isolation of mRNA and real-time PCR

Kidney tissue (10 mg) was homogenized in 500 µl RLT buffer reagent (Qiagen, Hilden, Germany) with an Ultraturrax for 30 s and total RNA was extracted with RNeasy® Mini columns (Qiagen) according to the manufacturer’s instructions. TaqMan reverse transcription reagents (Applied Biosystems, Weiterstadt, Germany) with random hexamers as primers were used to obtain first-strand cDNA. Final RNA concentration in the reaction mixture was adjusted to 0.1 ng/µl. To test for genomic DNA contamination, reactions without Multiscribe reverse transcriptase were performed as negative controls. Reverse transcription products were diluted 1:1 with dH_2_O. Subsequently, real-time PCR was accomplished with an ABI PRISM 7000 Sequence Detector System and SYBR Green (Applied Biosystems) or TaqMan reagents (Applied Biosystems) according to the manufacturer’s protocol. The relative amount of the specific mRNA was normalized with respect to 18S rRNA. See supplementary information (supplemental Table 1) for primers and probes used for amplification. All samples were run in triplicates. Primer pairs were designed using the Primer Express software (Perkin Elmer, Foster City, CA, USA).

### Analysis of data

Data are expressed as mean ± standard error of the mean (SEM). Student’s t-test was used to assess differences between UNX and their age-matched controls at the four weeks time point. To assess differences between UNX d1, UNX d14 and controls of the 52 weeks time point, first normality was tested using the Shapiro-Wilk test. One-way analysis of variance (one-way ANOVA), followed by Bonferroni *post hoc* test or, where appropriate, Kruskal-Wallis, followed by Dunn’s test were performed to assess significant differences between the groups using the GraphPad Prism software (Version 7, GraphPad Software Inc, San Diego, CA, USA). Results were considered significant at *p* < 0.05.

### Data availability

The datasets generated during and/or analysed during the current study are available from the corresponding author on reasonable request.

## Electronic supplementary material

Supplementary Information
