# Supplementary material for: Neonatal nephron loss during active nephrogenesis – detrimental impact with long-term renal consequences
Source: Sci Rep. 2018 Mar 14;8:4542. doi: 10.1038/s41598-018-22733-8 (PMC5852238; doi:10.1038/s41598-018-22733-8)

Neonatal nephron loss during active nephrogenesis - detrimental impact with long-term renal consequences

Carlos Menendez-Castro[#], Dörte Nitz[#], Nada Cordasic[§], Jutta Jordan[+], Tobias Bäuerle[+], Fabian B. Fahlbusch[#], Wolfgang Rascher[#], Karl F. Hilgers[§], Andrea Hartner[#]

Departments of [#]Pediatrics and Adolescent Medicine and [§]Nephrology and Hypertension, [+]Preclinical Imaging Platform Erlangen, Department of Radiology, University of Erlangen-Nurnberg, Erlangen, Germany

Supplemental Methods

Peri-interventional and surgical procedures of left-sided uninephrectomy

Pregnant dams were prepartally housed in individual cages. Left-sided uninephrectomy in pups was performed at day1 (UNX d1) or day 14 (UNX d14) of life. Animals were positioned on a heated blanket on the right side. All following steps of the surgery were performed under the microscope. Under isoflurane anesthesia and after disinfection of the surgical area intraperitoneal access was obtained by a left-sided flank incision of 7.5-10mm under aseptic conditions. The retroperitoneum was bluntly explored. After identification and careful separation of the left adrenal gland, the left kidney was mobilised and exenterated. Ureter and renal vessels were ligated by electrocautery and the left kidney was removed. The peritoneum was closed with single button sutures. For closure of the skin tissue adhesive (Histoacryl, B. Braun, Melsungen, Germany) was used. In sham operated animals the same surgical procedure was performed except for removal, the left kidney was only mobilized. For postinterventional recovery animals were left on the heated blanket until the return of spontaneous movements. Buprenorphine was applicated as a pre-emptive and postoperative analgetic in a dosage of 0.05-0.1mg/kg

Supplemental Table 1: List of primers pairs and probes used in the study.

	forward	reverse
**18S**	5'- TTG ATT AAG TCC CTG CCC TTT GT -3'	5'- CGA TCC GAG GGC CTC ACT A -3'
**α-SMA**	5'- TCCTGACCCTGAAGTATCCGATA -3'	5'- GGTGCCAGATCTTTTCCATGTC -3'
**CCL-2 (MCP-1)**	5'- CCTCCACCACTATGCAGGTCTC -3'<br>5'- TCACGCTTCTGGGCCTGTTGTTCA -3' [Probe]	5'- GCACGTGGATGCTACAGGC -3'
**CCL-3 (MIP-1α)**	5'- TCCTGCCACCTGCAAATCTC -3'	5'- GCTACTTGGCAGCAAACAGCT -3'
**CCL-5 (Rantes)**	5'- GTCGTCTTTGTCACTCGAAGGA -3'	5'- GATGTATTCTTGAACCCACTTCTTCTC -3'
**CCL-7 (MCP-3)**	5'- TGTGGGCCCAACCAGATG -3'	5'- CTTGGGAATCTTTTGTTTCTTGACA -3'
**CCR-2**	5'- CTGTGTGGTTGACATGCACTTAGA -3'<br>5'- AGACTCTTGGAATGACACACTGCTGCGTTA -3' [Probe]	5'- ACTCGGTCTGCTGTCTCCCTATAG -3'
**CD2AP**	5'- GATGAAAAATCACTGCTAGAACAGAAA -3'	5'- TTGGTAGGTGGAGTAGGCTTTTTG -3'
**Coll I**	5'- AGAGCGGAGAGTACTGGATCGA -3'	5'- CTGACCTGTCTCCATGTTGCA -3'
**Coll IV**	5'- AACGAAAGGGACACGAGGA -3'	5'- GGCCAGGAATACCAGGAAGT -3'
**FN**	5'- TTGCAACCCACCGTGGAGTATGTG -3'	5'- CTCGGTAGCCAGTGAGCTTAACAC -3'
**IFNγ**	5'- GCCAAGTTCGAGGTGAACAAC -3'<br>5'- CCAGCACAAAGCTGTCAATGAACTCATCA -3' [Probe]	5'- TAGATTCTGGTGACAGCTGGTGAA -3'
**IL-6**	5'- GCCCTTCAGGAACAGCTATGA -3'	5'- TGTCAACAACATCAGTCCCAAGA -3'
**KIM-1**	5'- ATAATCACACTGTAAGAATCCCTTTGAG -3'	5'- CAACGGACATGCCAACATAGA -3'
**MMP-2**	5'- CCCGACCAAGGATATAGCCTATT -3'	5'- CGGAAGTTCTTGGTGTAGGTGTAGA -3'
**Nephrin**	5'- GGGGACCCCTCTATGATGAA -3'	5'- GTGAAGCGTCTCACACCAGA -3'
**NEPH1**	5'- CCCCCTCCGGATCGAA -3'	5'- TGTCCCCACCTCTAGGAAGTTC -3'
**NGAL**	5'- TCACCCTGTACGGAAGAACCA -3'	5'- ACTTGGCAAAGCTGACGAATC -3'
**OPN**	5'- AAAGTGGCTGAGTTTGGCAG -3'	5'- AAGTGGCTACAGCATCTGAGTGT -3'

	5’- TCAGAGGAGAAGGCGCATTACAGCA -3’ [Probe]	
**PAI-1**	5‘- GTTCACCACTCCGGATGGG -3’	5’- TGGTAGGGCAGTTCCAGGAT -3’
**PECAM**	5’- TCCACGCCGGGAAGTACA -3’	5’- TCAATTGTGGTTTTTTCCTTGCT -3’
**Podocin**	5’- TCTTGTCCTCTCCTCCCTGA -3’	5’- AGACGGAGGTCAACCTTGTG -3’
**Renin**	5’- GCTACATGGAGAATGGGACTGAA -3’	5’- ACCACATCTTGGCTGAGGAAAC -3’
	5’- CCATCCACTATGGATCAGGGAAGGTCAA -3’ [Probe]	
**TGFβ-1**	5’- TGGAAGTGGATCCACGCGCCCAAGG -3’	5’- GCAGGAGCGCACGATCATGTTGGAC -3’
**TGFβ-2**	5’- CATCCCGCCCACTTTCTACA -3’	5’- ACATCGAAGCGGACGATTCT -3’
**TIMP-1**	5’- GATATGTCCACAAGTCCCAGAACC -3’	5’- CCACAGCCAGCACTATAGGTCTTT -3’
**TIMP-2**	5’- GCTGGACGTTGGAGGAAAGA -3’	5’- GCACAATAAAGTCACAGAGGGTAAT -3’
	5’- TCTCCTTCCGCCTTCCCTGCAATTAGA -3’ [Probe]	
**TNFα**	5’- ATGGGCTCCCTCTCATCAGT -3’	5’- GCTTGGTGGTTTGCTACGAC -3’
**Umod**	5’- TGCTCTTACCCTCTGGACATGA -3’	5’- AAGCTGATGTTCAAGGCACTAACC -3’
**VEGF-A**	5’- AACGAAAGCGCAAGAAATCC -3’	5’- GCTCACAGTGAACGCTCCAG -3’
**VEGF-R1**	5’- CGACACTCTTTTGGCTCCTTCTAAC -3’	5’- TGACAGGTAGTCCGTCTTTACTTCG -3’
**Vimentin**	5’- GAGTCAAACGAATACCGGAGACA -3’	5’- CCAGGGACTCATTAGTGCCTTT -3’

Supplemental Table 2: Markers of renal fibrosis and capillarisation at 52 weeks of age. n=8 for UNX d1, n=7 for UNX d14, n=8 for controls. Data are expressed as mean ± standard error of the mean. P-values were obtained from Bonferroni's post hoc-test following one-way ANOVA.

	**control**	**UNX d1**	**p-value UNX d1 vs control**	**UNX d14**	**p-value UNX d1 vs control**	**p-value UNXd1 vs UNXd14**
Tubuloint. Collagen IV expansion [%]	13.4±1.31	12.9±0.62	> 0.99	11.6±1.13	0.77	> 0.99
α-SMA-pos. interst. Cells [no/view]	59.5±3.42	55.0±3.63	> 0.99	55.9±4.07	> 0.99	> 0.99
Vimentin mRNA [fold induction]	1.00±0.08	1.65±0.34	0.23	1.68±0.26	0.22	> 0.99
α-SMA mRNA [fold induction]	1.00±0.07	1.24±0.27	> 0.99	1.20±0.08	> 0.99	> 0.99
Collagen IV mRNA [fold induction]	1.00±0.08	1.62±0.34	> 0.99	1.4±0.17	0.25	0.80
Fibronectin mRNA [fold induction]	1.00±0.10	2.31±0.62	0.10	1.56±0.17	0.72	0.51
PAI-1 mRNA [fold induction]	1.00±0.15	1 .58±0.63	0.88	0.94±0.09	> 0.99	0.79
TIMP-1 mRNA [fold induction]	1.00±0.16	2.27±0.57	**0.048**	1.88±0.32	0.16	0.50
TIMP-2 mRNA [fold induction]	1.00±0.13	1.13±0.21	0.93	1.07±0.09	0.98	0.99
MMP-2 mRNA [fold induction]	1.00±0.39	1.82±0.53	0.46	1.44±0.17	0.73	0.73
Tubuloint. RECA-pos. capillaries [no/view]	5.56±1.41	8.73±2.31	0.70	11.7±2.84	0.22	0.74
PECAM mRNA [fold induction]	1.00±0.13	0.69±0.05	0.09	0.74±0.08	0.23	> 0.99
VEGF-A mRNA [fold induction]	1.00±0.06	0.80±0.06	0.14	0.97±0.09	> 0.99	> 0.32
VEGF-R1 mRNA [fold induction]	1.00±0.10	0.90±0.10	> 0.99	1.07±0.16	> 0.99	> 0.99

Supplemental Table 3: Markers of renal inflammation at 52 weeks of age. n=8 for UNX d1, n=7 for UNX d14, n=8 for controls. Data are expressed as mean ± standard error of the mean. P-values were obtained from Bonferroni's post hoc-test following one-way ANOVA.

	**control**	**UNX d1**	**p-value UNX d1 vs control**	**UNX d14**	**p-value UNX d14 vs control**	**p-value UNX d1 vs UNX d14**
CCL2 (MCP-1) mRNA [fold induction]	1.00±0.18	1.89±0.58	0.26	0.88±0.12	0.99	0.25
CCL3 (MIP-1a) mRNA [fold induction]	1.00±0.21	0.99±0.27	> 0.99	1.03±0.17	> 0.99	> 0.99
CCL5 (Rantes) mRNA [fold induction]	1.00±0.19	0.95±0.11	0.99	0.78±0.09	0.63	0.78
CCL7 (MCP-3) mRNA [fold induction]	1.00±0.12	2.55±0.82	0.22	1.83±0.62	> 0.99	> 0.99
CCR2 mRNA [fold induction]	1.00±0.31	2.05±0.54	0.38	1.81±.0.49	> 0.99	0.73
Osteopontin mRNA [fold induction]	1.00±0.21	7.99±2.16	**0.04**	7.72±2.18	> 0.06	> 0.99
IL-6 mRNA [fold induction]	1.00±0.17	6.37±3.43	0.29	3.77±1.96	0.80	> 0.83
TNFα mRNA [fold induction]	1.00±0.13	1.39±0.33	0.64	1.23±0.24	0.91	0.96
TGF-β1 mRNA [fold induction]	1.00±0.07	1.75±0.42	0.25	1.56±0.24	0.51	0.96
TGF-β2 mRNA [fold induction]	1.00±0.10	1.68±0.30	0.27	1.59±0.36	0.41	0.99
IFNγ mRNA [fold induction]	1.00±0.39	1.06±0.15	> 0.99	1.01±0.18	> 0.99	> 0.99

Supplemental Figure 1

Exemplary photomicrographs of WT-1 (marker for podocytes) and RECA (marker for endothelial cells) stainings in renal sections of rats at the age of 52 weeks.

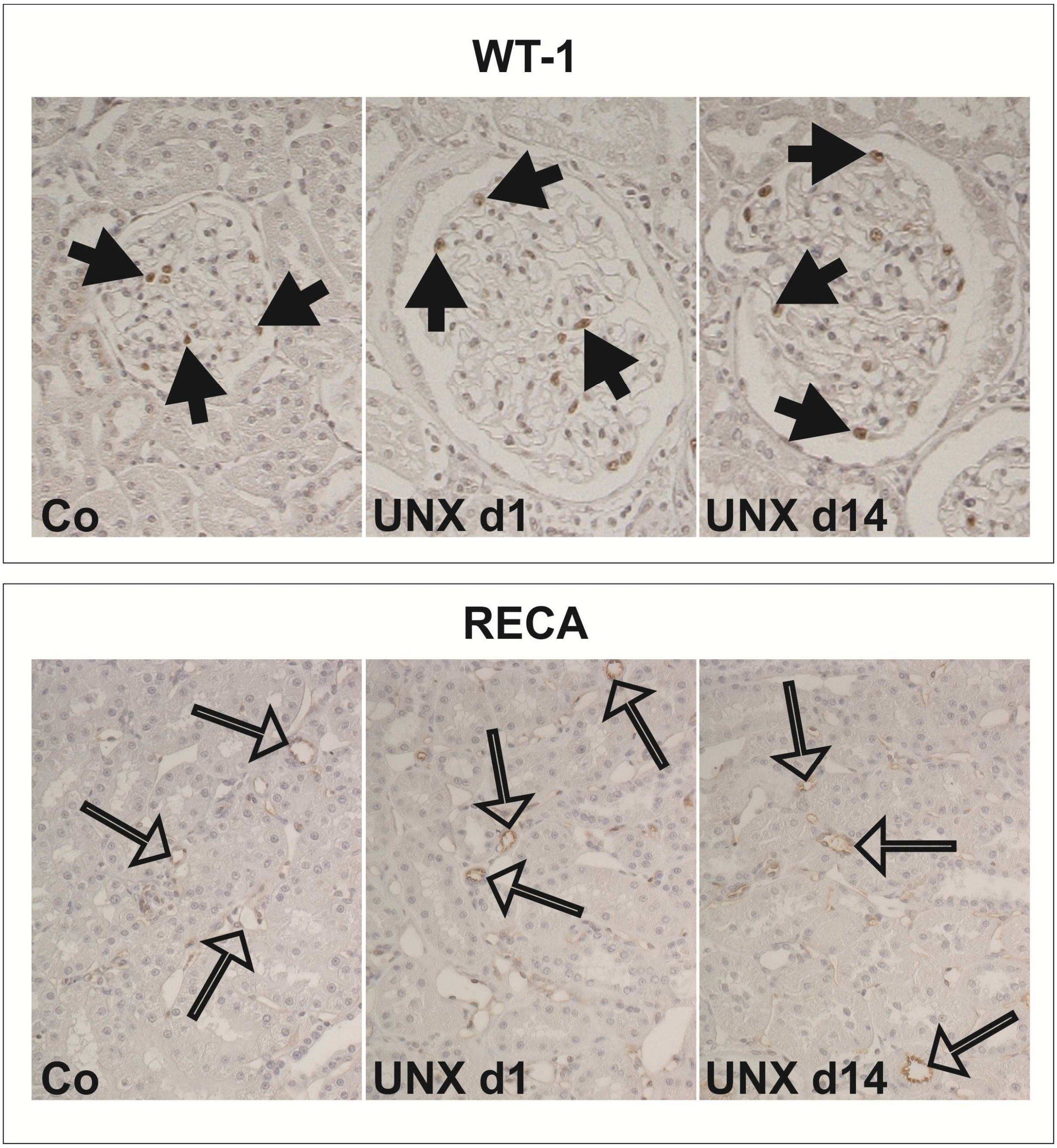

Supplemental Figure 2

A: Exemplary MR image of a glomerular ferritin labeled kidney before (left) and after (right) plugin assisted glomerular quantification. Glomerular labelling was obtained using cationized ferritin as described previously [1,2]. In short, rats obtained a fractional application of 3.3mg/100g cationized horse spleen ferritin (Sigma-Aldrich Chemicals, Munich, Germany) by 4 bolus injections in the tail vein. Retrograde perfusion was performed in deep anesthesia with NaCl 0.9% for 10 minutes and with paraformaldehyde for 5 minutes under a pressure of 200mmHg. Kidneys were weighted, the peri-renal capsules were removed and organs were embedded in 1.5% agarose and measured in a 7 Tesla Magnetic Resonance Tomograph (ClinScan, Bruker, Ettlingen, Germany) using a T1 weighted 3D Fast Low Angle Shot Sequence adapted from [1] with following parameters: time to repetition = 50 ms, time to echo = 7.8 ms, Flip angle = 15 °, 7 averages, acquisition time about 8 hours, base matrix = 704 x 704, field of view = 30 mm x 30 mm and a slice thickness of 0.13 mm.

B: Quantification of absolute glomerular numbers in glomerular ferritin labeled kidneys of rats after uninephrectomy at day 1 (UNX d1, n=5), day 14 (UNX d14, n=4) and day 42 (UNX d42, n=5) of life. A plugin of Chimaera GmbH (Erlangen, Germany) for Aycan Osirix Software (Aycan Digitalsysteme GmbH, Würzburg, Germany) was used to segment the hypointense glomeruli in contrast to the surrounding kidney tissue. Finally, the number of segmentated regions were counted as a measure for the number of glomeruli.

C: Exemplary photomicrograph of ferritin staining in a glomerular ferritin labeled kidney. As a control glomerular ferritin depositions were visualized with an antibody to ferritin (F6136, Sigma-Aldrich Chemicals, Munich, Germany) on frozen renal sections from ferritin-infused rats at a dilution of 1:1000.

References:

1 Chacon-Caldera, J. *et al.* Fast glomerular quantification of whole ex vivo mouse kidneys using Magnetic Resonance Imaging at 9.4 Tesla. *Z Med Phys* **26**, 54-62, doi:10.1016/j.zemedi.2015.12.008 (2016).

2 Bennett, K. M. *et al.* MRI of the basement membrane using charged nanoparticles as contrast agents. *Magnetic resonance in medicine* **60**, 564-574, doi:10.1002/mrm.21684 (2008).

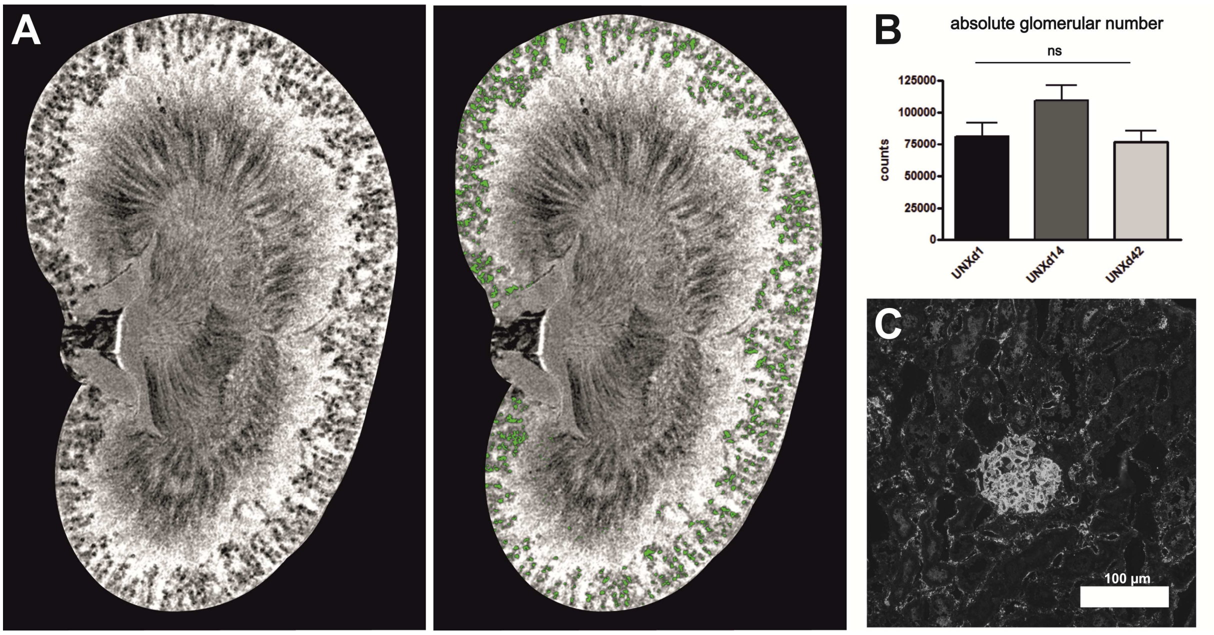

Supplement: Supplementary file 1 — Supplementary Information [file 41598_2018_22733_MOESM1_ESM.pdf]
